# The protective effectiveness of control interventions for malaria prevention: a systematic review of the literature

**DOI:** 10.12688/f1000research.12952.1

**Published:** 2017-11-01

**Authors:** Thomas Kesteman, Milijaona Randrianarivelojosia, Christophe Rogier

**Affiliations:** 1 Fondation Mérieux, Lyon, France; 2 Malaria Research Unit, Institut Pasteur de Madagascar, Antananarivo, Madagascar; 3 Ecole doctorale Sciences de la vie et de l’environnement, Université d’Antananarivo, Antananarivo, Madagascar; 4 Institute for Biomedical Research of the French Armed Forces (IRBA), Brétigny-Sur-Orge , France; 5 Unité de recherche sur les maladies infectieuses et tropicales émergentes - (URMITE), Marseille, France

**Keywords:** malaria, protective effectiveness, prevention and control, case-control studies, health surveys, insecticide-treated bed nets, indoor residual spraying, intermittent preventive treatment

## Abstract

**Background**: Thanks to a considerable increase in funding, malaria control interventions (MCI) whose efficacy had been demonstrated by controlled trials have been largely scaled up during the last decade. Nevertheless, it was not systematically investigated whether this efficacy had been preserved once deployed on the field. Therefore, we sought the literature to assess the disparities between efficacy and effectiveness and the effort to measure the protective effectiveness (PE) of MCI.

**Methods**: The PubMed database was searched for references with keywords related to malaria, to control interventions for prevention and to study designs that allow for the measure of the PE against parasitemia or against clinical outcomes.

**Results**: Our search retrieved 1423 references, and 162 articles were included in the review. Publications were scarce before the year 2000 but dramatically increased afterwards. Bed nets was the MCI most studied (82.1%). The study design most used was a cross-sectional study (65.4%). Two thirds (67.3%) were conducted at the district level or below, and the majority (56.8%) included only children even if the MCI didn’t target only children. Not all studies demonstrated a significant PE from exposure to MCI: 60.6% of studies evaluating bed nets, 50.0% of those evaluating indoor residual spraying, and 4/8 showed an added PE of using both interventions as compared with one only; this proportion was 62.5% for intermittent preventive treatment of pregnant women, and 20.0% for domestic use of insecticides.

**Conclusions**: This review identified numerous local findings of low, non-significant PE –or even the absence of a protective effect provided by these MCIs. The identification of such failures in the effectiveness of MCIs advocates for the investigation of the causes of the problem found. Ideal evaluations of the PE of MCIs should incorporate both a large representativeness and an evaluation of the PE stratified by subpopulations.

## Introduction

During the 2000s, several malaria control interventions have been largely adopted and scaled up in endemic countries. These interventions mainly include long lasting insecticidal nets (LLIN), indoor residual spraying (IRS), use of rapid diagnostic tests (RDT) to improve malaria diagnosis and artemisinin-based combination therapy (ACT) as first-line for the treatment of uncomplicated malaria, intermittent preventive treatment of pregnant women (IPTp), intermittent preventive treatment for infants (IPTi), and seasonal malaria chemoprevention (SMC). This considerable deployment of malaria control interventions has largely benefitted from the increase in international funding for the fight against malaria ^1^.

Before being scaled up, the efficacy of control interventions needs to be demonstrated in controlled trials (phase III). These trials consist generally in randomizing individuals or clusters, half receiving the intervention tested, and the other half receiving a control, i.e. a placebo or the best intervention available. Both the intervention and the control are strictly delivered under monitored conditions, in order to preclude biases in the estimate of the efficacy. For any given intervention, especially life-saving interventions, a small number of trials are conducted, because once the efficacy or the superiority of the intervention has been demonstrated it would be unethical to keep providing a less effective intervention to the population, and also because of the important resources needed to conduct such trials. These studies are indispensable to ensure that people receive best interventions available but they poorly depict what will be the effectiveness of the intervention once deployed in real life, for two reasons: (i) phase III trials are conducted in a limited number of settings that don’t encompass all possible field conditions, and (ii) special attention is paid in delivering the intervention during controlled trials, thus rendering ‘ideal’ conditions.

National malaria control programs and donors should nevertheless make sure that the effectiveness of the interventions is confirmed once they have been deployed on the field. As a matter of fact, detecting suboptimal effectiveness of control interventions is critical for policy guidance. This is becoming particularly important since the global budget of the fight against malaria ceased growing ^1^ and thus funding tends to be allocated for most effective interventions. The word “effectiveness” actually encompasses three concepts ^2, 3^: the coverage (does the intervention reach the population?), the individual protective effectiveness (does the intervention protect against or treat the disease?), and the community protective effectiveness (does the intervention benefit for others than the very ones who received the intervention?). Surveillance data and ecological studies can measure the overall impact of the control policy but they won’t be helpful in determining whether each intervention taken separately yielded the expected impact since all interventions are usually implemented concomitantly, and because they are influenced by environmental and social factors. Cross-sectional indicator surveys and social sciences studies will provide useful data regarding coverage of interventions and their determinants, but some data may be missing regarding the protective effectiveness (PE) of the interventions.

As mentioned above, it would be unethical - and laborious - to conduct controlled trials in all existing conditions and areas in order to verify the PE of malaria control interventions. Thus, alternative study designs must be applied. The PE can be evaluated (i) by biological studies measuring the bio-efficacy of drugs or insecticides (indirect measurement of the PE) or (ii) by epidemiological surveys yielding a direct measure of the PE of the intervention. The second approach encompasses three major study designs for phase IV assessment of the effectiveness of malaria control interventions, using historical non-compliant controls ^2^: case-control surveys (CCS), cross-sectional surveys (CSS), and cohorts. The stepped-wedge design also allows for the evaluation of the PE, although it sometimes relates to the efficacy (phase III) when the implementation of the intervention is strictly overseen, and sometimes to the effectiveness (phase IV) when the intervention is implemented under field conditions. All these study designs share as a common drawback the possibility of biases: non-compliant controls are not strictly comparable to people having received and using the intervention. Adjusting for socio-demographic variables can reduce but not eliminate biases at the individual level, and adjustment variables can be challenging to identify and to quantify when it comes to the evaluation of the community PE (ecological bias).

In order to assess the disparities between efficacy and effectiveness of malaria control interventions, and the range of PE observed on the field, we conducted a systematic review of the literature on epidemiological studies providing data about the protective effectiveness of control interventions for malaria prevention (CIMP) under field conditions. This study also has as secondary objectives (i) to appreciate what were the study designs most used, (ii) which populations were most surveyed, and (iii) what was the representativeness of these studies.

## Methods

The PubMed database was searched for references using an algorithm (provided in Supplementary File 1) looking for (i) keywords related to malaria and (ii) keywords related to control interventions for prevention and (iii) keywords related to study designs that allow for the measure of the effectiveness. Bibliographies of the articles identified were also examined to find additional reports. The search was run for the last time on June 23, 2015.

We intentionally excluded efficacy studies such as controlled trials in the context of phase III assessment, and studies that aimed at measuring indicators such as coverage or factors associated with the uptake of interventions. Studies aiming at measuring the bio-efficacy of interventions using other methods than epidemiological, were also excluded from the database. The present study focuses on intervention for malaria prevention: LLINs, IRS, IPTp, SMC, IPTi, larval source management, and information, education and communication (IEC) campaigns. Given that the use of other insecticides than IRS, such as repellents or mosquito coils, have recently demonstrated an interest in preventing malaria ^4– 6^, their PE were also recorded. Articles presenting the effectiveness of IEC regarding prevention behaviours were included. Only articles in English, French, Dutch, or Spanish were considered.

We focused on two outcomes: (i) the measure of the PE against peripheral parasitemia, as measured by RDT and/or blood smears and/or PCR, and (ii) the measure of the PE against occurrence of acute clinical malaria. In studies having investigated other biological or clinical outcomes simultaneously, we recorded preferentially the two outcomes mentioned above. Whenever several PE results were available from a single study (e.g. for different subpopulations), all PE results were retrieved.

On the basis of the objectives of the study disclosed in the title, the abstract and the article, we determined whether the study was aimed at measuring the effectiveness of a CIMP or if this measure was done “accidentally”, e.g. for the purpose of controlling for other associations. The PE was defined as one minus odds ratio (OR) or one minus relative risk (RR), depending on the study design.

## Results

Of the 1423 references retrieved, 523 were discarded on the basis of the title; 893 abstracts were checked and 683 of these didn't address the effectiveness of CIMP; seven abstracts could not be accessed (see flow diagram provided in Supplementary File 2). We thus identified 203 papers related to studies that aimed at measuring the effectiveness of CIMP or in which the effectiveness of CIMP was measured but 10 of them could not be accessed. One study was excluded because it focused on travellers and not resident populations of endemic areas; one reference was excluded because of the language; one study was excluded because it evaluated an intervention that was not particularly targeting malaria or mosquito control (cotrimoxazole in HIV positive pregnant women); two studies were excluded because it evaluated a CIMP out-dated (chloroquine chemoprophylaxis in pregnancy); 14 studies were excluded because no OR or RR value was presented and the data disclosed in the article didn’t allow for calculation of the PE of CIMP. Among the remaining 175 references, 13 presented methodological problems incompatible with the inclusion in the present review, such as absence of definition of cases or definition of the exposure to CIMP.

**Figure 1.**
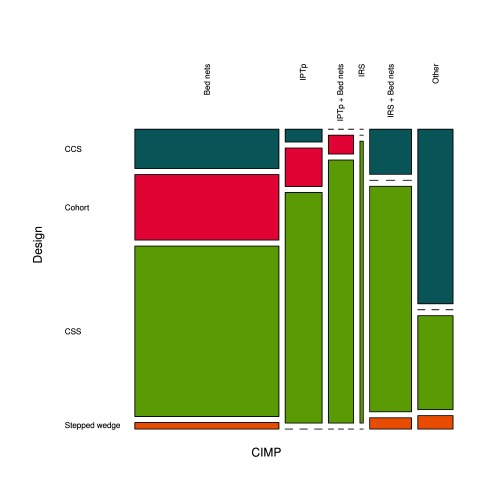
Proportions of CIMPs investigated in the 162 studies included in the review, and the proportions of study designs used.

The final review included thus 162 studies. This included 133 (82.1%) studies on bed nets, 37 (22.8%) studies on IPTp, 25 (15.4%) studies on IRS, and 22 (13.6%) studies on other interventions ( Figure 1). One third of the studies (52/162, 32.1%) addressed more than one CIMP. Regarding studies' design, 106 (65.4%) were CSS, 29 (17.9%) were CCS, 24 (14.8%) were cohorts, and 3 (1.9%) were stepped wedge ( Figure 1).

**Figure 2.**
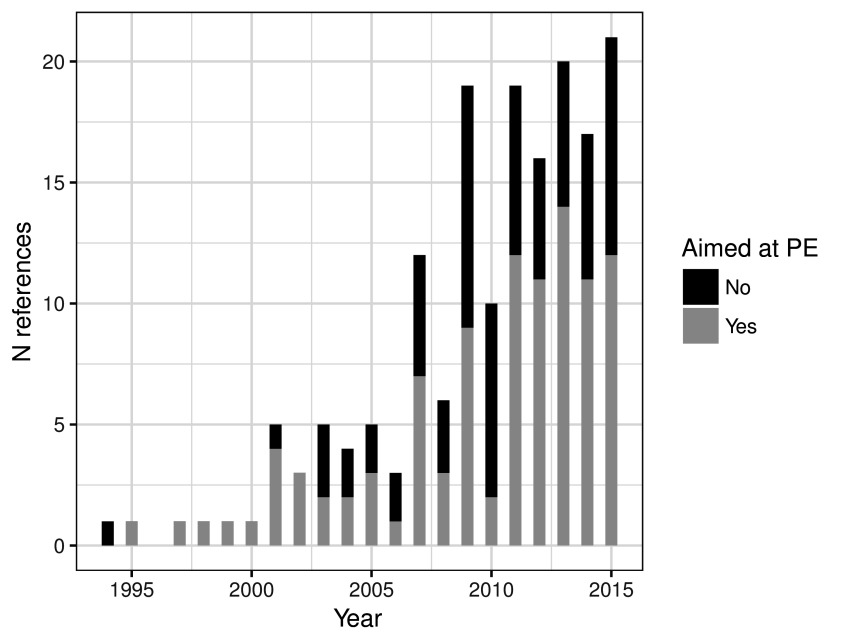
Number of references by year of publication, and the proportion of studies whose aim was the measure of the effectiveness of CIMP. The last year is truncated since the search was performed in June 2015.

The number of publications related to the PE of CIMP increased considerably during the years 2000’s and stagnated since 2010 ( Figure 2). Two fifths (41.4%) of these studies were not directly aimed at measuring the PE of CIMP.

Regarding the representativeness of the studies, two thirds (109/162, 67.3%) were conducted at the district level or below. Only one third of the studies that didn’t investigate the effectiveness of IPTp (43/125, 34.4%) included the whole population while the majority (71/125, 56.8%) included only children. Only 15 of these studies (12.0%) were conducted in the whole population and at a regional level (≥2 districts, province/region, or island) or above (national or multi-country).

In 18.1% (50/276) of the evaluations of PE retrieved or recalculated from the 162 studies included in the review, the association between the malaria outcome and the exposure to the CIMP was not adjusted on other variables (univariate logistic regression, two-by-two tables, etc). Since the adjustment on age is particularly important, we calculated the proportion of studies conducted in a population where the age of the oldest participants was ≥10 years older than the youngest, but for which no adjustment on age was done in the measure of the PE. We found that 38.9% of the studies conducted in such a population with heterogeneous age groups didn’t adjust the calculation of the PE for age.

### PE of bed nets

**Figure 3.**
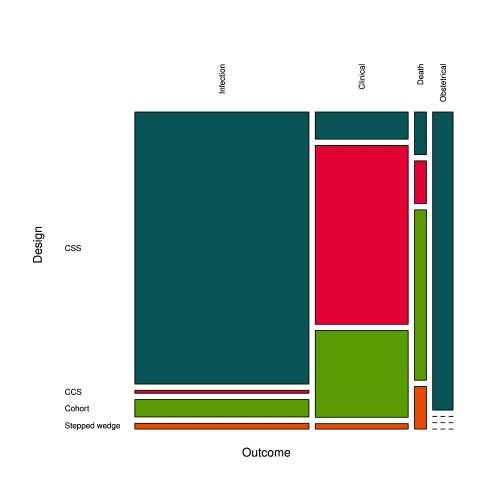
Proportions of study designs and outcomes investigated for the evaluation of PE of bed nets.

The search retrieved 169 measures of the PE of bed nets in 133 studies ( Supplementary File 3). Most of the time (82.2%), the exposure to bed nets was measured at the individual level, but in 23 cases (13.6%) it was done at household level (ownership or proportion of users) and in seven cases (4.1%) at cluster level. The majority of PE measurements involved insecticide-treated nets (ITN) or LLINs (42.0 and 12.4%, respectively) but in an important proportion of cases the definition of bed nets didn’t include impregnation or not (42.6%). In some instances (2.9%), the measurement was specifically done for non-impregnated bed nets (NIBN). Most PE evaluations used the *Plasmodium* infection as outcome (56.8%), especially CSS that accounted for 62.7% of study designs ( Figure 3), or clinical malaria (31.9%), especially CCS that accounted for 20.1% of study designs; some used an obstetrical outcome (7.1%), and a few ones used the mortality as outcome (4.1%). Cohorts represented 15.4% of study designs and there were only three stepped-wedge designs (1.8%). More than a half of PE results (58.0%) were obtained from paediatric populations and 27.8% considered the whole population; the other studies (14.2%) were conducted on women of childbearing age.

**Figure 4.**
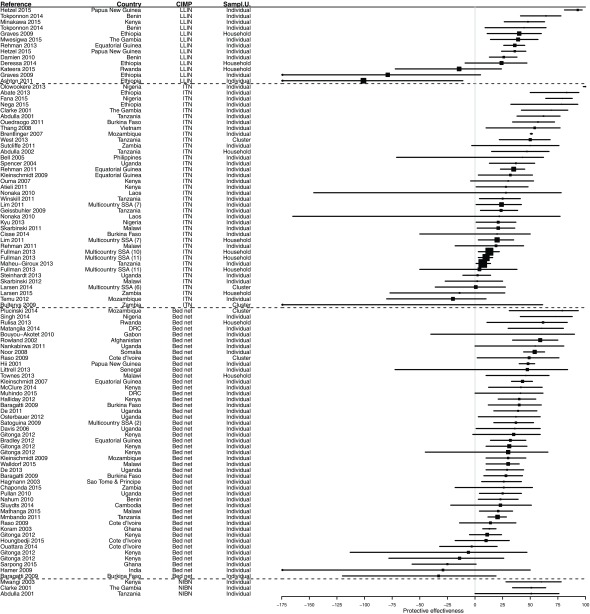
Forest plot of evaluations of PE of bed nets against infection. Results without CI are not displayed. Box size is proportional to the sample size.

**Figure 5.**
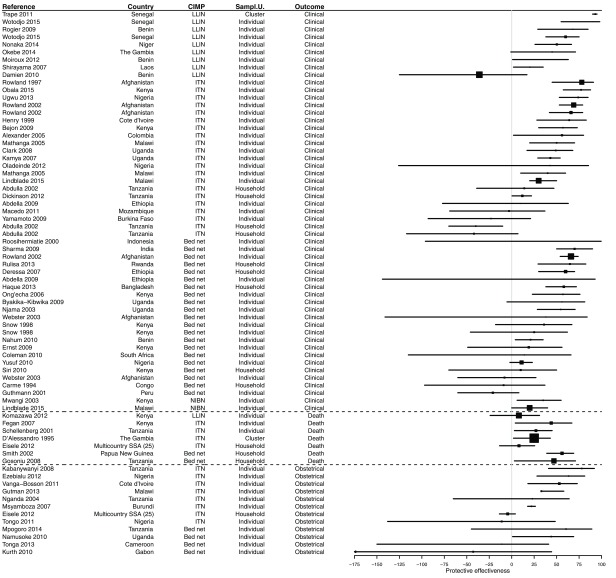
Forest plot of evaluations of PE of bed nets against other outcomes than infection. Results without CI are not displayed. Box size is proportional to the sample size.

Most of the results (60.7%) demonstrated a significant PE from bed nets use. However, 38.2% of results were not significant ( Figure 4, Figure 5 and Supplementary File 3). In 14.2% of cases, the PE value was negative, i.e. a trend towards a risk increased, and 1.2% of results showed a risk significantly increased. The median PE was 36.0% (interquartile range [IQR] 14.0–54.0%), and this differed only marginally according to CIMP definition: median PE was 39.8% (IQR 20.2–50.3%) for LLINs, 30.0% (IQR 16.5–52.0%) for ITNs, 51.0% (IQR 35.0–51.0%) for NIBNs, and 36.0 (IQR 13.8–54.6%) for bed nets without further precision of their impregnation.

### PE of IRS

The search retrieved 32 measures of the PE of IRS in 25 studies ( Supplementary File 4). CSS survey design was largely predominant (90.6%) and three PE evaluations from CCS (9.4%) were observed. A third of studies (34.4%) considered the whole population while 59.4% were obtained from paediatric populations and 6.3% from women of childbearing age. Most of the time (78.1%), the exposure to bed nets was measured at the household level, but in seven cases (21.9%) it was measured at cluster level. Only 21.9% of PE measurements of IRS considered recent spraying (≤6 months before the survey or delay since last IRS round in months), and the rest considered IRS ‘last round’, ‘last year’, or even ‘ever’. Most PE evaluations used the *Plasmodium* infection as outcome (87.5%) and the rest considered clinical malaria (12.5%).

**Figure 6.**
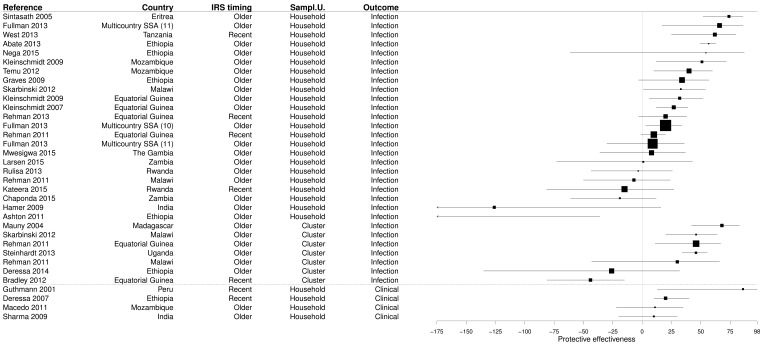
Forest plot of evaluations of PE of IRS. Box size is proportional to the sample size. Recent spraying: ≤6 months before the survey or delay since last IRS round in months.

Half of results demonstrated a significant PE of IRS (median 28.5%, IQR 8.8–47.3%), but 43.8% of results were not significant and 6.2% of results showed a risk significantly increased ( Figure 6 and Supplementary File 4). The PE value was positive in more than three results out of four (78.1%). Median PEs were comparable when considering recent (20.0%, IQR -2.5–41.0%) or older spraying (32.0%, IQR 9.0–46.0%).

### PE of concurrent exposure to ITN and IRS

Our systematic search allowed us identifying only five studies and eight results about the PE of concurrent exposure to ITN and IRS ( Table 1). Two results compared the exposure to both interventions versus IRS only, and the six other compared the exposure to both interventions versus no intervention. All study designs were CSS and all evaluated the effectiveness of ITN or LLIN against infection in children.

**Table 1.** PE of the exposure to IRS and bed nets in decreasing order, by reference group for PE measurement. *: Indicates significant result as compared with exposure to one CIMP only. MC: Multi-country study stratified by transmission (low-medium-high), the number between brackets indicates the number of countries. †: PE versus IRS only. ‡: PE versus no ITN and no IRS.

Country	PE of ITN + IRS (%) [95%CI]	PE ^‡^ of ITN ( Supplementary File 2)	PE ^‡^ of IRS ( Supplementary File 3)	Reference
**Mozambique**	37 [21;50] * ^†^	30 [10;46] *	51 [12;72] *	Kleinschmidt 2009 ^31^
**Equatorial Guinea**	35 [2;57] * ^†^	36 [26;45] *	20 [-3;38]	Rehman 2013 ^11^
**Equatorial Guinea**	29 [14;41] * ^†^	32 [3;52] *	32 [6;52] *	Kleinschmidt 2009 ^31^
**MC (10) – Med. transm.**	53 [37;67] * ^‡^	13 [3;22] *	20 [3;34] *	Fullman 2013 ^42^
**Mozambique**	50 [30;70] ^‡^	-20 [-80;10]	40 [10;60] *	Temu 2012 ^46^
**MC (11) – Low transm.**	33 [-33;70] ^‡^	4 [-50;38]	66 [17;86] *	Fullman 2013 ^42^
**MC (11) – High transm.**	31 [11;47] ^‡^	10 [3;16] *	9 [-30;36]	Fullman 2013 ^42^
**Malawi**	19 [-19;44] ^‡^	2 [-27;25]	33 [1;54] *	Skarbinski 2012 ^45^

Four out of eight results demonstrated a significant added PE of using both interventions as compared with one of these two CIMP only; in these studies (or sub-studies) ITN and IRS alone had both demonstrated significant PE –the PE of IRS in the study of Rehman *et al.* is borderline. In the other four (sub-)studies, one of the two CIMP had failed to demonstrate a significant PE and the exposure to both interventions either showed an added protection but non-significant as compared with one CIMP only or provided a PE inferior to the PE of IRS only.

### PE of IPTp

Our search retrieved 40 measures of the PE of IPTp using sulfadoxine-pyrimethamine (SP) in 37 studies ( Supplementary File 5). Among these 40 results, 16 (40.0%) compared any regimen versus no SP dose, 13 (32.5%) compared the standard regimen versus no IPTp, and the remaining 11 (27.5%) compared the standard regimen versus substandard regimen. Most PE evaluations used an obstetrical or neonatal (e.g. low birth weight) outcome only (45.0%) or an outcome considering an obstetrical event or a maternal peripheral parasitemia (7.5%). The detection of *Plasmodium* in the mother’s blood was used as the outcome in 37.5%; three results (7.5%) had evaluated clinical malaria, and one used the mortality as outcome (2.5%). CSS represented 85.0% of study designs, cohorts 10.0% and there were only two CCS (5.0%). Most results (90.0%) were obtained from mothers, usually pregnant women at antenatal consultation and/or women at delivery units, but 10.0% considered paediatric populations (neonates or infants). The vast majority of studies were conducted at the district level or below (85.0%).

**Figure 7.**
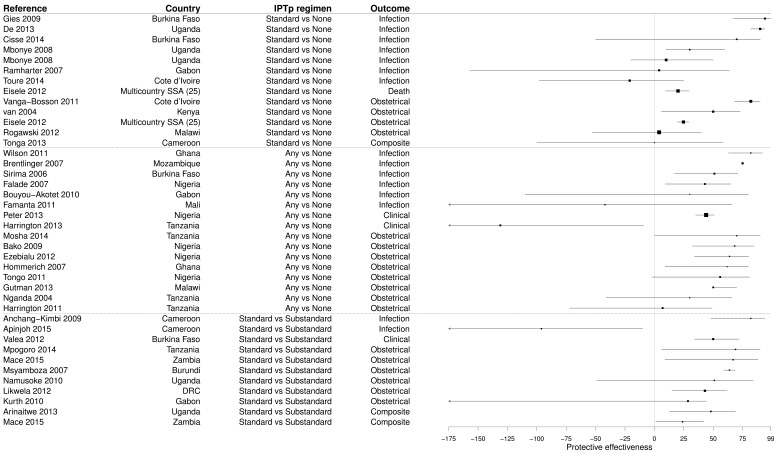
Forest plot of evaluations of PE of IPTp. Box size is proportional to the sample size.

Most results demonstrated a significant PE of IPTp (median PE 49.0%, IQR 23.0–67.3%), but 32.5% of results were not significant and 5.0% of results showed a risk significantly increased ( Figure 7 and Supplementary File 5). Median PE was 24.7% (IQR 4.0–70.0%) in studies evaluating standard IPTp regimen versus no IPTp, 50.5% (IQR 30.0–65.0%) in studies comparing any IPTp regimen versus no IPTp, and 50.0% (IQR 35.8–65.4%) in studies evaluating standard IPTp regimen versus substandard IPTp regimen. These values were comparable between studies evaluating the PE of IPTp against infection (median PE 43.0, IQR 7.0–78.5%) and studies evaluating the PE of IPTp against obstetrical outcomes (median PE 53.5, IQR 33.3–66.2%).

### PE of the domestic use of insecticides

Our systematic search identified 20 evaluations, from 16 studies ( Supplementary File 6), of the PE of the use of other insecticides than IRS, including coils (45.0%), sprays (30.0%), and repellents (10.0%). In the remaining 15.0% of cases, the PE of two or three of these formulations together was evaluated. The vast majority of these results came from CCS evaluating clinical outcomes (15/20, 80.0%), and the four other results came from CSS evaluating clinical (1/20) infection (1/20), or obstetrical (2/20) outcomes. The majority of these studies were conducted in paediatric populations (65.0%), some in the whole population (20.0%) and the remaining 15.0% among adult women.

**Figure 8.**
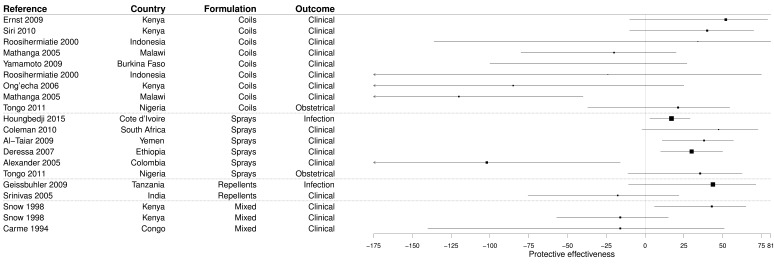
Forest plot of evaluations of PE of domestic use of insecticides. Box size is proportional to the sample size.

Overall the PE of these insecticides was demonstrated in only four studies, whatever the formulation in coils, sprays, or repellents, and most (70.0%) results were non-significant ( Figure 8 and Supplementary File 6). The median PE was 19.1% (IQR -21.0–38.5%).

### Other interventions

Our search retrieved only one study aiming to evaluate the PE of IPTi. It was a CCS conducted among infants in Tanzania and its main result was that the PE against occurrence of clinical malaria cases was 18% and not significant (95% CI -129–71% ^165^).

We identified two studies evaluating the PE of larviciding programs by comparing clusters receiving the intervention and clusters that were not treated, either in a CSS design applied in under-fives ^38^ or in a stepped-wedge design encompassing all age groups ^43^. Both studies showed a significant PE of larviciding against infection by *Plasmodium* of 72% (20–90%) and 21% (7–34%) respectively.

In this review, no study assessing the PE of IEC interventions on malaria indicators has been found, but we found two countrywide CSS evaluating the effectiveness of the exposure to IEC programs on bed net (or ITN) use. One was conducted in adult population of Cameroon ^166^ and the other one in adult women of Zambia ^167^. These two studies showed that being exposed to IEC interventions was associated with an increase in bed net use (OR 1.48, 95% CI 1.18–1.86, and OR 1.62, 95% CI 1.28–2.04, respectively) by logistic regression with propensity score matching.

Finally, we found one study having evaluated the PE of availability of a village health worker trained for malaria management against *Plasmodium* infection through a CSS conducted among all age groups in a province of the Philippines ^27^. They found a significant PE of 74% (16–92%).

Data underlying the results presented in this systematic review Click here for additional data file. Copyright: © 2017 Kesteman T et al. 2017 Data associated with the article are available under the terms of the Creative Commons Attribution Licence, which permits unrestricted use, distribution, and reproduction in any medium, provided the original data is properly cited.

## Discussion

This review showed that the efforts made for the evaluation of the effectiveness is increasing with time, in parallel with the global funding available for malaria control. Nevertheless, the number of published studies about the effectiveness of CIMPs seems to be stagnating since 2010. This could hinder the progress towards more cost-effective control policies, as the strategy should be locally adapted depending on data about the effectiveness of CIMP.

Overall, there is a sense of a low representativeness of the studies. Only one third of the studies were conducted at a large scale, and only one third included all ages and genders; only one out of eight had both features. Several CIMPs target the whole population of a region or a country, e.g. IRS or universal distributions of LLINs. When evaluating the PE of such CIMP, it’s crucial not to leave aside a part of the population since the effectiveness of CIMP may vary depending on transmission or between age groups for example ^67, 168, 169^. On the contrary, nearly 40% of studies conducted across age groups didn’t include the age in regression models while age influences both malaria outcomes (e.g. probability to be infected) and CIMP coverage ^170^. In order to yield unbiased evaluation of the PE of a CIMP, it is critical to adjust the measure of the association of malaria outcome and exposure to CIMP for age, as well as for other variables known to influence the outcome (e.g. socio-economic status, parity, rural or urban area) and to take into account the intra-cluster correlation in multi-stage sample designs. Moreover, several studies conducted at a large scale didn’t stratify the analysis. Since local features of malaria transmission or cultural behaviours may affect (or enhance) the PE of CIMPs, omitting stratified analysis precludes the identification of clusters where the effectiveness of CIMPs was suboptimal.

On the other hand, the multiplicity of local evaluations of the PE of CIMP offers an appreciation of the diversity of local conditions. Certain studies revealed that the PE was largely above what had been demonstrated in efficacy trials; this can result from biases inherent to observational studies, but it’s also possible that local conditions favour the effectiveness of CIMP, e.g. the PE of LLINs is expected to be especially high where vector populations exclusively bite indoor and late at night. Conversely, many studies failed to demonstrate the PE of CIMP studied or showed that it was lower than expected. This is where the interest of these surveys stands, for it urges policy makers and their research partners to investigate the causes of this failure and to propose alternative control interventions. This is also why we didn’t conduct a meta-analysis on the data retrieved in this review. Besides this, various meta-analyses of CIMP already exist, either reviewing efficacy studies only ^169, 168, 171^, or mixed both efficacy and effectiveness studies ^172^.

This review presents several limitations, including the search in one database only, the limits in languages considered (although one reference only was discarded for this reason), and the incomplete access to articles. This review is thus probably not exhaustive but it intends to be largely representative of effectiveness studies. On purpose, we didn’t include meta-analyses of the studies included as the overall objective was to get a sense of what kind of studies had been done, and to be strictly descriptive on the results obtained. Our take-home message is not so much that MCI are effective on average, but that their effectiveness might be locally lower -or higher- than what is expected by efficacy studies.

### Bed nets

Overall, the measures of the PE of bed nets demonstrated a fair effectiveness of this CIMP, even often above the protective efficacy measured in controlled trials. This phenomenon can be attributed to local features of malaria transmission (e.g. intensity of transmission, vector biting behaviour, vector sensitivity to insecticides, or human behaviour), or to differences in outcomes used in efficacy trials (often clinical outcomes) versus those used in effectiveness studies (often the infection by *Plasmodium* parasites), or to differences in the definition of the exposure to bed nets. For example, it has been shown that in low transmission areas LLIN perform better and/or parasitemia is a better indicator of LLIN performance ^37, 168^. Controlled trials performed in areas of high transmission (e.g. two meta-analyses of studies conducted in such areas showed protective efficacies of 13% ^168^ and 17% ^173^, respectively) have shown lower protective efficacy than those conducted in low transmission areas, e.g. in Kenyan highlands (protective efficacy 63%, ^174^) or in Pakistan (protective efficacy 43%, ^175^).

Various definitions of bed net exposure have been used throughout the studies included in the present review: type of bed net (LLIN, ITN, bed net without further definition of impregnation but sometimes in areas where most bed nets are actually impregnated, or NIBN), intensity of exposure (ownership, bed net/person ratio, use the previous night, or regular use), and level of measure of exposure (individual, household, cluster). Surprisingly, in our review, it seems that the definition of exposure to bed nets does not impacts importantly the measure of the PE. Therefore, it is possible that evaluations of the PE of LLINs or ITNs yield an estimation of the effectiveness provided by the physical barrier against vectors’ bites and underestimate the community effect offered by insecticides impregnation.

### IRS

Effectiveness studies generally verified the PE of IRS, whether at community or household level. It’s complicated to compare those results with efficacy studies since those are relatively scarce. Indeed, IRS has been deployed before the requirement of a demonstration of efficacy of MCI through randomized controlled trials. A meta-analysis from 13 efficacy and effectiveness studies conducted in 11 countries measured a pooled household-level and community-level protective efficacy of 62% ^172^, but other controlled trials showed more limited protective efficacy at the community level, e.g. in India where it was 28% ^169, 176^ and in Nigeria during the wet season where it was 26% ^169, 177^. Controlled trials even sometimes showed very limited efficacy like in Nigeria during the dry season or in Tanzania (protective efficacy 6% ^169, 177, 178^).

As for bed nets, the vectors’ biting behaviour, their sensitivity to insecticides, and the endemicity of malaria are expected to influence most the PE of IRS. These factors should be investigated in areas where IRS fails to demonstrate its effectiveness in order to guide local malaria control policies.

### Concurrent exposure to LLIN and IRS

Overall effectiveness studies plea for the combination of these two vector control interventions since it seems that all studies finding significant PE of the two CIMP separately also found a significant added PE in people benefitting from both CIMP simultaneously. On the contrary, in studies where at least one CIMP failed to demonstrate a significant PE, the added value of using LLIN in a household having received IRS also failed to be proven. Results from randomized controlled trials are more balanced: some did show an additional protection offered by IRS over LLIN only ^179, 180^, some did not ^181– 183^. Overall, evidence of additional protection of the combination against malaria remains inconclusive ^184^.

### IPTp

Most studies aiming to evaluate the effectiveness of IPTp were conducted at small scale, usually in one or two hospitals. Nevertheless, some studies were conducted at a larger scale and even stratified by regions, e.g. a study conducted in 3 regions of the Democratic Republic of Congo showed that, in one regions, the effectiveness of IPTp against low birth weight was affected while it was preserved in the 2 other regions ^161^. Geographical stratification can thus detect an inhomogeneity in the PE that can reflect, for example, local parasitological resistance to SP. This resistance is the major cause to be investigated for policy guidance.

An important limitation of the present review is that IPTp aims at reducing malaria burden in terms of maternal and neonatal morbidity and mortality; obstetrical outcomes are therefore more adapted for the evaluation of the PE of IPTp than maternal peripheral parasitemia or acute clinical episodes of malaria –that we prioritized in our review. Similarly these outcomes were often considered as secondary in efficacy controlled trials. Nevertheless one meta-analysis of three studies conducted in two countries measured a pooled protective efficacy of IPTp against maternal peripheral parasitemia of 55% ^185^ and another trial demonstrated a protective efficacy of 64% ^186^. Besides this, the efficacy of IPTp has been evaluated against several obstetrical outcomes, including low birth weight (significant protective efficacy of 29% ^185^), placental parasitemia (significant protective efficacy of 52% ^185^), maternal anaemia (significant protective efficacy of 10% ^185^), perinatal mortality (non-significant protective efficacy of 22% ^187^), or stillbirth (non-significant protective efficacy of 4% ^187^).

### Other interventions

The PE of the use of insecticides was seldom demonstrated, despite the possibility of a socioeconomic bias that would be expected to increase their PE. Further studies will have to be conducted in order to verify that their efficacy translate into effectiveness under field conditions if they are adopted by policy makers.

Our review identified no study having tried to evaluate the effectiveness of IEC interventions against clinical or biological malaria indicators, and only two that demonstrated the effectiveness of IEC programs on bed net coverage. Unfortunately, the uniqueness of media messages and cultural features in these studies preclude the extrapolation of their results. Generally few studies have evaluated the effectiveness of IEC intervention, not only for malaria ^188^. This reflects the rarity of phase III studies aiming at demonstrating the efficacy of IEC interventions on epidemiological indices. This paucity of information is surprising given the popularity of IEC programs in public health.

In 2013, IPTi had been adopted by one country only ^1^, which explains that we found only one study evaluating its PE. More surprisingly, SMC has been adopted by six countries ^1^ but the PE of this CIMP has not been evaluated yet. The small number of studies regarding SMC, IEC or larviciding hinders the interpretation of these results.

## Conclusions

This review shows that there is an increasing interest in measuring the PE of CIMPs. Most studies confirmed the PE of the CIMPs that they were evaluating, but an important part yielded a ‘negative’ PE and/or non-significant confidence interval. In this case, complementary investigations are needed in order to confirm the existence of a problem in the effectiveness of the CIMP and to propose alternative control measures if necessary.

A frequent feature of the studies included in this review was the low geographical representativeness and/or the low representativeness in the population studied. Conversely the analyses of large samples were not systematically stratified by subpopulations. We believe that such investigations need to zoom out (encompass a large population) and to zoom in (stratify by subpopulations) to get a complete picture evaluating the effectiveness of CIMPs.

To evaluate properly the PE of a CIMP we recommend to pay attention to the following points: (i) encompass all age groups and genders, except for targeted interventions such as IPTp or IPTi, (ii) sample all geographical and/or cultural patterns, (iii) stratify the evaluation of the PE by subgroups, (iv) adjust for socio-demographic variables that are associated with the outcomes and at least adjust for age and gender if the population sampled is not homogeneous in this regard.

## Abbreviations

ACT: artemisinin-based combination therapy, CCS: case-control survey, CSS: cross-sectional survey, CIMP: control intervention for malaria prevention, IEC: information, education and communication, IPTi: intermittent preventive treatment for infants, IPTp: intermittent preventive treatment of pregnant women, IQR: interquartile range, ITN: insecticide-treated nets, IRS: indoor residual spraying, LLIN: long lasting insecticidal nets, NIBN: non-impregnated bed nets, RDT: rapid diagnostic tests, SMC: seasonal malaria chemoprevention; SP: sulfadoxine-pyrimethamine

## Data availability

The data referenced by this article are under copyright with the following copyright statement: Copyright: © 2017 Kesteman T et al.

Data associated with the article are available under the terms of the Creative Commons Attribution Licence, which permits unrestricted use, distribution, and reproduction in any medium, provided the original data is properly cited. 

**Dataset 1: Data underlying the results presented in this systematic review.** DOI, 10.5256/f1000research.12952.d182415 ^189^.

## Supplementary material

**Supplementary File 1: Algorithm for the systematic review of the literature.**

**Supplementary File 2: PRISMA flow diagram.**

**Supplementary File 3: PE of bed nets.**

**Supplementary File 4: PE of IRS.**

**Supplementary File 5: PE of IPTp.**

**Supplementary File 6: PE of domestic use of insecticides.**

**Supplementary File 7: PRISMA checklist.**
