# Supplementary material for: The protective effectiveness of control interventions for malaria prevention: a systematic review of the literature
Source: F1000Res. 2017 Nov 1;6:1932. [Version 1] doi: 10.12688/f1000research.12952.1 (PMC5721947; doi:10.12688/f1000research.12952.1)
Supplement: Supplementary file 2 [file f1000research-6-14045-s0001.tgz › be60d052-fd35-47fe-a36e-5253b8746733.pdf]

Supplementary File 1 – Algorithm for the systematic review of the literature.

Concept

Malaria (1) AND control interventions for prevention (2) AND study design for the evaluation of the effectiveness (3):

1. “malaria” OR “Plasmodium”
2. “mosquito control” OR “mosquito net” OR “bed net” OR “Long Lasting Insecticidal Net” OR “insecticide treated bed net” OR “indoor residual spraying” OR “house-spraying” OR “larval control” OR “larval source management” OR “seasonal malaria chemoprophylaxis” OR “intermittent preventive treatment” OR “IEC” OR “BCC”
3. “Case-Control Studies” OR “Risk Factors” OR “Odds Ratio” OR “Prospective Studies” OR “effectiveness” OR “Cross-Sectional Studies” OR “Cohort Studies”

Code

(“malaria” [Mesh] OR “malaria” [TIAB] OR “Plasmodium” [Mesh] OR “Plasmodium” [TIAB]) AND (“mosquito Nets”[Mesh] OR “mosquito control”[Mesh] OR “mosquito net”[TIAB] OR “bed net”[TIAB] OR “bednet”[TIAB] OR “Long Lasting Insecticidal Net” [TIAB] OR “insecticide-treated bed net”[TIAB] OR “insecticide treated bed net”[TIAB] OR “insecticide-treated net”[TIAB] OR “insecticide treated net”[TIAB] OR “Insecticide-Treated Bednets”[Mesh] OR “mosquito control”[TIAB] OR “indoor residual spraying”[TIAB] OR “house-spraying”[TIAB] OR “house spraying”[TIAB] OR “larval source management”[TIAB] OR “larval control”[TIAB] OR “seasonal malaria chemoprophylaxis”[TIAB] OR “seasonal malaria chemoprevention”[TIAB] OR “intermittent preventive treatment”[TIAB] OR “IPTp”[TIAB] OR “IPTc”[TIAB] OR (“information”[TIAB] AND “education”[TIAB] AND “communication”[TIAB]) OR ((“behaviour”[TIAB] OR “behavior” [TIAB]) AND “change”[TIAB] AND “communication”[TIAB]) OR “IEC”[TIAB] OR “BCC”[TIAB] OR “IEC/BCC”[TIAB]) AND (“Case-Control Studies”[Mesh] OR “Case-Control”[TIAB] OR (“Case”[TIAB] AND “Control”[TIAB]) OR “risk factor”[TIAB] OR “Risk Factors”[Mesh] OR “Odds Ratio”[Mesh] OR “Odds Ratio”[TIAB] OR “Prospective Studies”[Mesh] OR “effectiveness”[TIAB] OR “cross-sectional”[TIAB] OR “Cross-Sectional Studies”[Mesh] OR “cohort”[TIAB] OR “Cohort Studies”[Mesh])
