# Supplementary figures and images for: The protective effectiveness of control interventions for malaria prevention: a systematic review of the literature
Source: F1000Res. 2017 Nov 1;6:1932. [Version 1] doi: 10.12688/f1000research.12952.1 (PMC5721947; doi:10.12688/f1000research.12952.1)

Supplementary File 2 – PRISMA flow diagram

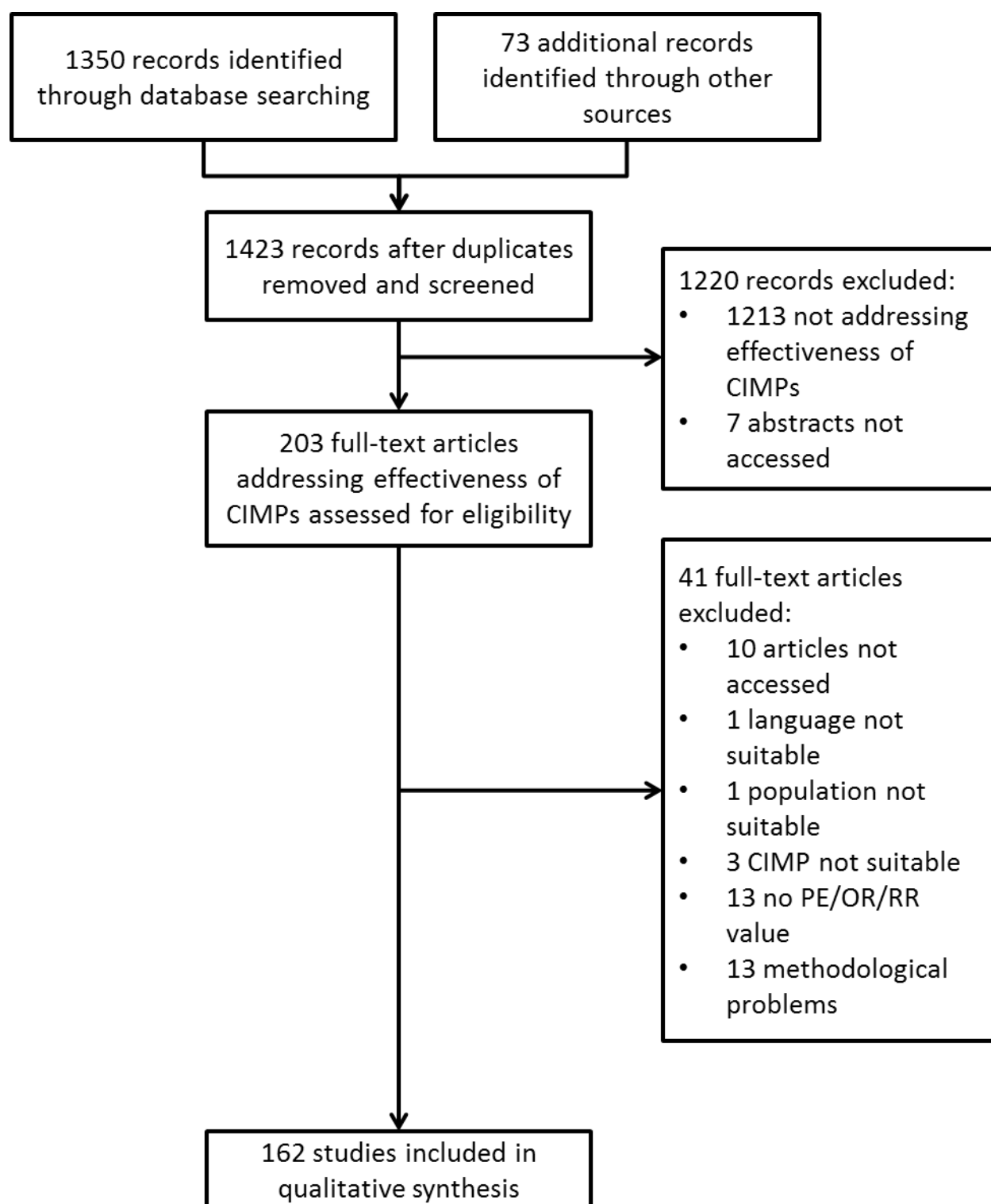

Supplement: Supplementary file 3 [file f1000research-6-14045-s0002.tgz › 5026f4ea-8f51-4c53-85f0-ede4de1763a1.pdf]
