# Supplementary material for: The protective effectiveness of control interventions for malaria prevention: a systematic review of the literature
Source: F1000Res. 2017 Nov 1;6:1932. [Version 1] doi: 10.12688/f1000research.12952.1 (PMC5721947; doi:10.12688/f1000research.12952.1)
Supplement: Supplementary file 4 [file f1000research-6-14045-s0003.tgz › 614b353d-98b5-43a6-a3c3-b7b6bb74ab0b.pdf]

**Supplementary File 3 – PE of bed nets.** PE of bed nets in decreasing order, by outcome measured, and bed net definition. *: indicates significant result. MC: multi-country.†: in multi-country studies the number between brackets indicates the number of countries. ITN: insecticide-treated nets. LLIN: long lasting insecticidal nets. NIBN: non-impregnated bed nets. CCS: case-control survey. CSS: cross-sectional survey.

| Outcome | CIMP | Sampling unit | Study design | Country† | PE (%) [95%CI] | Reference |
|---|---|---|---|---|---|---|
| **Infection** | LLIN | Individual | CSS | Benin | 64 [41;78]* | Tokponnon 2014 [7] |
| **Infection** | LLIN | Individual | CSS | Kenya | 48 [26;63]* | Minakawa 2015 [8] |
| **Infection** | LLIN | Individual | CSS | Benin | 44 [9;64]* | Tokponnon 2014 [7] |
| **Infection** | LLIN | Household | CSS | Ethiopia | 40 [11;60]* | Graves 2009 [9] |
| **Infection** | LLIN | Individual | CSS | The Gambia | 39 [14;57]* | Mwesigwa 2015 [10] |
| **Infection** | LLIN | Individual | CSS | Equatorial Guinea | 36 [26;45]* | Rehman 2013 [11] |
| **Infection** | LLIN | Individual | Cohort | Benin | 26 [13;38]* | Damien 2010 [12] |
| **Infection** | LLIN | Household | CSS | Ethiopia | 24 [-9;47] | Deressa 2014 [6] |
| **Infection** | LLIN | Household | CSS | Rwanda | -14 [-72;24] | Kateera 2015 [13] |
| **Infection** | LLIN | Individual | CSS | Ethiopia | -79 [-236;5] | Graves 2009 [9] |
| **Infection** | LLIN | Individual | CSS | Ethiopia | -101 [-264;-11]* | Ashton 2011 [14] |
| **Infection** | ITN | Individual | Cohort | Nigeria | 100 [98;100]* | Olowookere 2013 [15] |
| **Infection** | ITN | Individual | CSS | Ethiopia | 83 [50;94]* | Abate 2013 [16] |
| **Infection** | ITN | Individual | CSS | Nigeria | 79 [64;88]* | Fana 2015 [17] |
| **Infection** | ITN | Individual | CSS | Ethiopia | 78 [32;93]* | Nega 2015 [18] |
| **Infection** | ITN | Individual | CSS | The Gambia | 69 [42;84]* | Clarke 2001 [19] |
| **Infection** | ITN | Individual | CSS | Tanzania | 62 [38;77]* | Abdulla 2001 [20] |
| **Infection** | ITN | Individual | CSS | Burkina Faso | 57 [34;72]* | Ouédraogo 2011 [21] |
| **Infection** | ITN | Individual | CSS | Vietnam | 54 [10;77]* | Thang 2008 [22] |
| **Infection** | ITN | Individual | CSS | Mozambique | 51 [50;52]* | Brentlinger 2007 [23] |
| **Infection** | ITN | Cluster | CSS | Tanzania | 50 [22;68]* | West 2013 [24] |
| **Infection** | ITN | Individual | CSS | Zambia | 50 [-3;76] | Sutcliffe 2011 [25] |
| **Infection** | ITN | Household | CSS | Tanzania | 47 [15;67]* | Abdulla 2002 [26] |
| **Infection** | ITN | Individual | CSS | Philippines | 43 [-71;62] | Bell 2005 [27] |
| **Infection** | ITN | Individual | CSS | DRC | 41 | Maketa 2015 [28] |
| **Infection** | ITN | Individual | CSS | Uganda | 37 [13;54]* | Spencer 2004 [29] |

| Outcome | CIMP | Sampling unit | Study design | Country[†] | PE (%) [95%CI] | Reference |
|---|---|---|---|---|---|---|
| **Infection** | ITN | Individual | CSS | Equatorial Guinea | 35 [23;45]* | Rehman 2011 [30] |
| **Infection** | ITN | Individual | CSS | Equatorial Guinea | 32 [3;52]* | Kleinschmidt 2009 [31] |
| **Infection** | ITN | Individual | CSS | Kenya | 30 [-4;53] | Ouma 2007 [32] |
| **Infection** | ITN | Individual | CSS | Kenya | 28 [1;48]* | Atieli 2011 [33] |
| **Infection** | ITN | Individual | CSS | Laos | 28 [-146;78] | Nonaka 2010 [34] |
| **Infection** | ITN | Individual | CSS | Tanzania | 25 [4;41]* | Winskill 2011 [35] |
| **Infection** | ITN | Cluster | Step. wedge | The Gambia | 25 | D'Alessandro 1995 [36] |
| **Infection** | ITN | Individual | CSS | Multicountry (7) | 24 [1;42]* | Lim 2011 [37] |
| **Infection** | ITN | Individual | CSS | Tanzania | 24 [5;39]* | Geissbühler 2009 [38] |
| **Infection** | ITN | Individual | CSS | Laos | 22 [-165;77] | Nonaka 2010 [34] |
| **Infection** | ITN | Individual | CSS | Nigeria | 21 [1;37]* | Kyu 2013 [39] |
| **Infection** | ITN | Individual | CSS | Malawi | 21 [2;36]* | Skarbinski 2011 [40] |
| **Infection** | ITN | Individual | CSS | Burkina Faso | 20 [-50;50] | Cisse 2014 [41] |
| **Infection** | ITN | Household | CSS | Multicountry (7) | 20 [3;35]* | Lim 2011 [37] |
| **Infection** | ITN | Individual | CSS | Malawi | 19 [-18;44] | Rehman 2011 [30] |
| **Infection** | ITN | Household | CSS | MC (10) – Med.T. | 13 [3;22]* | Fullman 2013 [42] |
| **Infection** | ITN | Household | CSS | MC (11) – High T. | 10 [3;16]* | Fullman 2013 [42] |
| **Infection** | ITN | Individual | CSS | Tanzania | 7 [1;14]* | Maheu-Giroux 2013 [43] |
| **Infection** | ITN | Household | CSS | MC (11) – Low T. | 4 [-50;38] | Fullman 2013 [42] |
| **Infection** | ITN | Individual | CSS | Uganda | 2 [-11;14] | Steinhardt 2013 [44] |
| **Infection** | ITN | Individual | CSS | Malawi | 2 [-27;25] | Skarbinski 2012 [45] |
| **Infection** | ITN | Individual | CSS | Mozambique | -20 [-80;10] | Temu 2012 [46] |
| **Infection** | ITN | Cluster | CSS | Zambia | -150 [-1567;61] | Bulterys 2009 [47] |
| **Infection** | Bed net | Cluster | CSS | Mozambique | 79 [31;94]* | Plucinski 2014 [48] |
| **Infection** | Bed net | Individual | CSS | Nigeria | 73 [41;88]* | Singh 2014 [49] |
| **Infection** | Bed net | Household | CSS | Rwanda | 62 [11;84]* | Rulisa 2013 [50] |
| **Infection** | Bed net | Individual | CSS | DRC | 60 [30;80]* | Matangila 2014 [51] |
| **Infection** | Bed net | Individual | CSS | Gabon | 60 [-40;90] | Bouyou-Akotet 2010 [52] |
| **Infection** | Bed net | Individual | CSS | Afghanistan | 59 [34;75]* | Rowland 2002 [53] |
| **Infection** | Bed net | Individual | Cohort | Uganda | 55 [0;80]* | Nankabirwa 2011 [54] |
| **Infection** | Bed net | Individual | CSS | Somalia | 54 [44;63]* | Noor 2008 [55] |

| Outcome | CIMP | Sampling unit | Study design | Country[†] | PE (%) [95%CI] | Reference |
|---|---|---|---|---|---|---|
| **Infection** | Bed net | Cluster | CSS | Cote d'Ivoire | 49 [2;76]* | Raso 2009 [56] |
| **Infection** | Bed net | Individual | CSS | Papua N. Guinea | 48 [40;54]* | Hii 2001 [57] |
| **Infection** | Bed net | Individual | CSS | Senegal | 47 [-72;84] | Littrell 2013 [58] |
| **Infection** | Bed net | Household | CSS | Malawi | 46 [10;67]* | Townes 2013 [59] |
| **Infection** | Bed net | Individual | CSS | Equatorial Guinea | 43 [33;52]* | Kleinschmidt 2007 [60] |
| **Infection** | Bed net | Individual | Cohort | Kenya | 42 [12;61]* | McClure 2014 [61] |
| **Infection** | Bed net | Individual | CCS | DRC | 41 [0;62]* | Muhindo 2015 [62] |
| **Infection** | Bed net | Individual | CSS | Kenya | 40 [21;55]* | Halliday 2012 [63] |
| **Infection** | Bed net | Individual | CSS | Burkina Faso | 40 [12;60]* | Baragatti 2009 [64] |
| **Infection** | Bed net | Individual | CSS | Uganda | 39 [17;56]* | De Beaudrap 2011 [65] |
| **Infection** | Bed net | Individual | CSS | Uganda | 37 [3;59]* | Osterbauer 2012 [66] |
| **Infection** | Bed net | Individual | CSS | Multicountry (2) | 37 [17;53]* | Satoguina 2009 [67] |
| **Infection** | Bed net | Individual | CSS | Uganda | 36 [1;59]* | Davis 2006 [68] |
| **Infection** | Bed net | Individual | CSS | Kenya | 35 [-2;59] | Gitonga 2012 [69] |
| **Infection** | Bed net | Individual | CSS | Equatorial Guinea | 32 [14;46]* | Bradley 2012 [70] |
| **Infection** | Bed net | Individual | CSS | Kenya | 31 [10;47]* | Gitonga 2012 [69] |
| **Infection** | Bed net | Individual | CSS | Kenya | 30 [-45;66] | Gitonga 2012 [69] |
| **Infection** | Bed net | Individual | CSS | Mozambique | 30 [10;46]* | Kleinschmidt 2009 [31] |
| **Infection** | Bed net | Individual | Cohort | Uganda | 29 [10;44]* | De Beaudrap 2013 [71] |
| **Infection** | Bed net | Individual | CSS | Burkina Faso | 28 [8;43]* | Baragatti 2009 [64] |
| **Infection** | Bed net | Individual | CSS | Sao Tome & Pr. | 26 [6;42]* | Hagmann 2003 [72] |
| **Infection** | Bed net | Individual | CSS | Uganda | 25 [4;42]* | Pullan 2010 [73] |
| **Infection** | Bed net | Individual | CSS | Benin | 23 [3;39]* | Nahum 2010 [74] |
| **Infection** | Bed net | Individual | CSS | Cambodia | 23 [-22;51] | Sluydts 2014 [75] |
| **Infection** | Bed net | Individual | CSS | Tanzania | 20 [12;28]* | Mmbando 2011 [76] |
| **Infection** | Bed net | Individual | CSS | Cote d'Ivoire | 14 [-14;37] | Raso 2009 [56] |
| **Infection** | Bed net | Individual | CSS | Ghana | 13 [7;19]* | Koram 2003 [77] |
| **Infection** | Bed net | Individual | CSS | Kenya | 11 [-5;24] | Gitonga 2012 [69] |
| **Infection** | Bed net | Individual | CSS | Cote d'Ivoire | 10 [-18;31] | Houngbedji 2015 [78] |
| **Infection** | Bed net | Individual | CSS | Tanzania | 5 | Somi 2007 [79] |
| **Infection** | Bed net | Individual | Cohort | Cote d'Ivoire | -3 [-32;20] | Ouattara 2014 [80] |

| Outcome | CIMP | Sampling unit | Study design | Country[†] | PE (%) [95%CI] | Reference |
|---|---|---|---|---|---|---|
| **Infection** | Bed net | Individual | CSS | Kenya | -6 [-113;47] | Gitonga 2012 [69] |
| **Infection** | Bed net | Individual | CSS | Kenya | -14 [-78;26] | Gitonga 2012 [69] |
| **Infection** | Bed net | Individual | CSS | Ghana | -25 [-57;1] | Sarpong 2015 [81] |
| **Infection** | Bed net | Individual | CSS | India | -29 [-229;50] | Hamer 2009 [82] |
| **Infection** | Bed net | Individual | CSS | Burkina Faso | -33 [-120;19] | Baragatti 2009 [64] |
| **Infection** | NIBN | Individual | CSS | Kenya | 60 [28;78]* | Mwangi 2003 [83] |
| **Infection** | NIBN | Individual | CSS | The Gambia | 51 [34;64]* | Clarke 2001 [19] |
| **Infection** | NIBN | Individual | CSS | Tanzania | 51 [0;76]* | Abdulla 2001 [20] |
| **Clinical** | LLIN | Cluster | Cohort | Senegal | 93 [90;95]* | Trape 2011 [84] |
| **Clinical** | LLIN | Individual | CCS | Senegal | 90 [55;98]* | Wotodjo 2015 [85] |
| **Clinical** | LLIN | Individual | CCS | Benin | 68 [29;85]* | Rogier 2009 [86] |
| **Clinical** | LLIN | Individual | Cohort | Senegal | 60 [38;75]* | Wotodjo 2015 [87] |
| **Clinical** | LLIN | Individual | CSS | Niger | 50 [26;67]* | Nonaka 2014 [88] |
| **Clinical** | LLIN | Individual | CCS | The Gambia | 45 [-1;71] | Okebe 2014 [89] |
| **Clinical** | LLIN | Individual | Cohort | Benin | 40 [1;63]* | Moiroux 2012 [90] |
| **Clinical** | LLIN | Individual | CSS | Laos | 20 [2;35]* | Shirayama 2007 [91] |
| **Clinical** | LLIN | Individual | Cohort | Benin | -36 [-125;17] | Damien 2010 [12] |
| **Clinical** | ITN | Individual | CCS | Afghanistan | 78 [45;91]* | Rowland 1997 [92] |
| **Clinical** | ITN | Individual | Cohort | Nigeria | 74 [53;85]* | Ugwu 2013 [93] |
| **Clinical** | ITN | Individual | CCS | Afghanistan | 69 [53;79]* | Rowland 2002 [53] |
| **Clinical** | ITN | Individual | CCS | Afghanistan | 66 [42;79]* | Rowland 2002 [53] |
| **Clinical** | ITN | Individual | Step. wedge | Cote d'Ivoire | 64 [28;83]* | Henry 1999 [94] |
| **Clinical** | ITN | Individual | Cohort | Kenya | 57 [30;73]* | Bejon 2009 [95] |
| **Clinical** | ITN | Individual | CCS | Colombia | 56 [2;80]* | Alexander 2005 [96] |
| **Clinical** | ITN | Individual | CCS | Malawi | 50 [20;70]* | Mathanga 2005 [97] |
| **Clinical** | ITN | Individual | Cohort | Uganda | 49 [17;68]* | Clark 2008 [98] |
| **Clinical** | ITN | Individual | Cohort | Uganda | 43 [29;54]* | Kamya 2007 [99] |
| **Clinical** | ITN | Individual | CCS | Nigeria | 43 [-126;86] | Oladeinde 2012 [100] |
| **Clinical** | ITN | Individual | CCS | Malawi | 40 [10;60]* | Mathanga 2005 [97] |
| **Clinical** | ITN | Individual | Cohort | Ethiopia | 39 | Loha 2012 [101] |
| **Clinical** | ITN | Individual | Cohort | Malawi | 30 [20;50]* | Lindblade 2015 [102] |

| Outcome | CIMP | Sampling unit | Study design | Country† | PE (%) [95%CI] | Reference |
|---|---|---|---|---|---|---|
| **Clinical** | ITN | Household | CCS | Tanzania | 14 [-39;47] | Abdulla 2002 [26] |
| **Clinical** | ITN | Household | CSS | Tanzania | 12 [0;22]* | Dickinson 2012 [103] |
| **Clinical** | ITN | Individual | CCS | Ethiopia | 10 [-77;63] | Abdella 2009 [104] |
| **Clinical** | ITN | Individual | CCS | Mozambique | -3 [-69;37] | Macedo de Oliv 2011 [105] |
| **Clinical** | ITN | Individual | CCS | Burkina Faso | -23 [-93;21] | Yamamoto 2009 [106] |
| **Clinical** | ITN | Household | Cohort | Tanzania | -40 [-70;-10]* | Abdulla 2002 [26] |
| **Clinical** | ITN | Household | CCS | Tanzania | -42 [-117;7] | Abdulla 2002 [26] |
| **Clinical** | Bed net | Individual | CCS | Indonesia | 91 [-96;100] | Roosihermiatie 2000 [107] |
| **Clinical** | Bed net | Individual | CCS | India | 70 [50;90]* | Sharma 2009 [108] |
| **Clinical** | Bed net | Individual | CCS | Afghanistan | 66 [54;74]* | Rowland 2002 [53] |
| **Clinical** | Bed net | Household | CCS | Rwanda | 65 [29;82]* | Rulisa 2013 [50] |
| **Clinical** | Bed net | Household | CSS | Ethiopia | 60 [30;70]* | Deressa 2007 [109] |
| **Clinical** | Bed net | Individual | CCS | Ethiopia | 59 [-144;93] | Abdella 2009 [104] |
| **Clinical** | Bed net | Household | Cohort | Bangladesh | 58 [38;72]* | Haque 2013 [110] |
| **Clinical** | Bed net | Individual | CCS | Kenya | 57 [23;76]* | Ong'echa 2006 [111] |
| **Clinical** | Bed net | Individual | CCS | Uganda | 56 [-5;81] | Byakika-Kibw. 2009 [112] |
| **Clinical** | Bed net | Individual | Cohort | Uganda | 55 [29;71]* | Njama 2003 [113] |
| **Clinical** | Bed net | Individual | CCS | Afghanistan | 38 [-141;84] | Webster 2003 [114] |
| **Clinical** | Bed net | Individual | CCS | Kenya | 36 [-18;67] | Snow 1998 [115] |
| **Clinical** | Bed net | Individual | CCS | Kenya | 25 [-46;62] | Snow 1998 [115] |
| **Clinical** | Bed net | Individual | Cohort | Benin | 21 [4;35]* | Nahum 2010 [74] |
| **Clinical** | Bed net | Individual | CCS | Kenya | 19 [-49;56] | Ernst 2009 [116] |
| **Clinical** | Bed net | Individual | CCS | South Africa | 14 [-115;66] | Coleman 2010 [117] |
| **Clinical** | Bed net | Individual | CSS | Nigeria | 11 [-2;23] | Yusuf 2010 [118] |
| **Clinical** | Bed net | Household | CCS | Kenya | 10 [-70;50] | Siri 2010 [119] |
| **Clinical** | Bed net | Individual | CCS | Afghanistan | -8 [-60;27] | Webster 2003 [114] |
| **Clinical** | Bed net | Household | CCS | Congo | -9 [-97;37] | Carme 1994 [120] |
| **Clinical** | Bed net | Individual | CCS | Peru | -21 [-60;8] | Guthmann 2001 [121] |
| **Clinical** | Bed net | Individual | CCS | Indonesia | -83615 | Roosihermiatie 2000 [107] |
| **Clinical** | NIBN | Individual | Cohort | Kenya | 35 [6;55]* | Mwangi 2003 [83] |
| **Clinical** | NIBN | Individual | Cohort | Malawi | 20 [0;40]* | Lindblade 2015 [102] |

| Outcome | CIMP | Sampling unit | Study design | Country† | PE (%) [95%CI] | Reference |
|---|---|---|---|---|---|---|
| **Death** | LLIN | Individual | Cohort | Kenya | 8 [-24;31] | Komazawa 2012 [122] |
| **Death** | ITN | Individual | Cohort | Kenya | 44 [4;67]* | Fegan 2007 [123] |
| **Death** | ITN | Individual | CCS | Tanzania | 27 [3;45]* | Schellenberg 2001 [124] |
| **Death** | ITN | Cluster | Step. wedge | The Gambia | 25 [2;43]* | D'Alessandro 1995 [36] |
| **Death** | ITN | Household | CSS | Multicountry (25) | 8 [-14;26] | Eisele 2012 [125] |
| **Death** | Bed net | Household | Cohort | Papua N. Guinea | 56 [39;69]* | Smith 2002 [126] |
| **Death** | Bed net | Household | Cohort | Tanzania | 47 [3;71]* | Gosoniu 2008 [127] |
| **Obstetrical** | ITN | Individual | CSS | Tanzania | 78 [41;92]* | Kabanywanyi 2008 [128] |
| **Obstetrical** | ITN | Individual | CSS | Nigeria | 63 [28;81]* | Ezebialu 2012 [129] |
| **Obstetrical** | ITN | Individual | CSS | Cote d'Ivoire | 53 [18;73]* | Vanga-Bosson 2011 [130] |
| **Obstetrical** | ITN | Individual | CSS | Malawi | 33 [33;58]* | Gutman 2013 [131] |
| **Obstetrical** | ITN | Individual | CSS | Tanzania | 23 [-65;64] | Nganda 2004 [132] |
| **Obstetrical** | ITN | Individual | CSS | Burundi | 22 [18;26]* | Msyamboza 2007 [133] |
| **Obstetrical** | ITN | Household | CSS | Multicountry (25) | -4 [-14;4] | Eisele 2012 [125] |
| **Obstetrical** | ITN | Individual | CSS | Nigeria | -11 [-138;48] | Tongo 2011 [134] |
| **Obstetrical** | Bed net | Individual | CSS | Tanzania | 60 [-45;89] | Mpogoro 2014 [135] |
| **Obstetrical** | Bed net | Individual | CSS | Uganda | 44 [1;69]* | Namusoke 2010 [136] |
| **Obstetrical** | Bed net | Individual | CSS | Cameroon | -11 [-150;41] | Tonga 2013 [137] |
| **Obstetrical** | Bed net | Individual | CSS | Gabon | -43 [-233;44] | Kurth 2010 [138] |
