# Supplementary material for: The protective effectiveness of control interventions for malaria prevention: a systematic review of the literature
Source: F1000Res. 2017 Nov 1;6:1932. [Version 1] doi: 10.12688/f1000research.12952.1 (PMC5721947; doi:10.12688/f1000research.12952.1)
Supplement: Supplementary file 5 [file f1000research-6-14045-s0004.tgz › 5461f690-f20a-4cfb-a646-f3688adf6981.pdf]

Supplementary File 4 – PE of IRS. PE of IRS in decreasing order, by outcome measured, and level of evaluation of IRS exposure (household versus cluster). *: indicates significant result. MC: multi-country. †: in multi-country studies the number between brackets indicates the number of countries. CCS: case-control survey. CSS: cross-sectional survey. IRS timing: recent spraying means ≤6 months before the survey or delay since last IRS round in months.

Outcome	IRS timing	Sampling unit	Study design	Country†	PE (%) [95%CI]	Reference
**Infection**	Older	Household	CSS	Eritrea	74 [52;86]*	Sintasath 2005 [139]
**Infection**	Older	Household	CSS	MC (11) – Low T.	66 [17;86]*	Fullman 2013 [42]
**Infection**	Recent	Household	CSS	Tanzania	62 [25;80]*	West 2013 [24]
**Infection**	Older	Household	CSS	Ethiopia	57 [49;63]*	Abate 2013 [16]
**Infection**	Older	Household	CSS	Ethiopia	54 [-61;87]	Nega 2015 [18]
**Infection**	Older	Household	CSS	Mozambique	51 [12;72]*	Kleinschmidt 2009 [31]
**Infection**	Older	Household	CSS	Mozambique	40 [10;60]*	Temu 2012 [46]
**Infection**	Older	Household	CSS	Ethiopia	34 [-3;57]	Graves 2009 [9]
**Infection**	Older	Household	CSS	Malawi	33 [1;54]*	Skarbinski 2012 [45]
**Infection**	Older	Household	CSS	Equatorial Guinea	32 [6;52]*	Kleinschmidt 2009 [31]
**Infection**	Older	Household	CSS	Equatorial Guinea	27 [12;39]*	Kleinschmidt 2007 [60]
**Infection**	Recent	Household	CSS	Equatorial Guinea	20 [-3;38]	Rehman 2013 [11]
**Infection**	Older	Household	CSS	MC (10) – Med. T.	20 [3;34]*	Fullman 2013 [42]
**Infection**	Recent	Household	CSS	Equatorial Guinea	10 [-1;20]	Rehman 2011 [30]
**Infection**	Older	Household	CSS	MC (11) – High T.	9 [-30;36]	Fullman 2013 [42]
**Infection**	Older	Household	CSS	The Gambia	8 [-36;37]	Mwesigwa 2015 [10]
**Infection**	Older	Household	CSS	Rwanda	-3 [-44;26]	Rulisa 2013 [50]
**Infection**	Older	Household	CSS	Malawi	-7 [-50;24]	Rehman 2011 [30]
**Infection**	Recent	Household	CSS	Rwanda	-15 [-82;27]	Kateera 2015 [13]
**Infection**	Older	Household	CSS	India	-126 [-510;16]	Hamer 2009 [82]
**Infection**	Older	Household	CSS	Ethiopia	-191 [-521;-36]*	Ashton 2011 [14]
**Infection**	Older	Cluster	CSS	Madagascar	68 [42;83]*	Mauny 2004 [140]
**Infection**	Older	Cluster	CSS	Malawi	46 [20;64]*	Skarbinski 2012 [45]
**Infection**	Older	Cluster	CSS	Equatorial Guinea	46 [11;67]*	Rehman 2011 [30]
**Infection**	Older	Cluster	CSS	Uganda	46 [34;56]*	Steinhardt 2013 [44]
**Infection**	Older	Cluster	CSS	Malawi	30 [-43;66]	Rehman 2011 [30]

Outcome	IRS timing	Sampling unit	Study design	Country[†]	PE (%) [95%CI]	Reference
**Infection**	Older	Cluster	CSS	Ethiopia	-26 [-135;32]	Deressa 2014 [6]
**Infection**	Recent	Cluster	CSS	Equatorial Guinea	-44 [-81;-15]*	Bradley 2012 [70]
**Clinical**	Recent	Household	CCS	Peru	86 [13;98]*	Guthmann 2001 [121]
**Clinical**	Recent	Household	CSS	Ethiopia	20 [10;40]*	Deressa 2007 [109]
**Clinical**	Older	Household	CCS	Mozambique	11 [-22;35]	Macedo de Oliv 2011 [105]
**Clinical**	Older	Household	CCS	India	10 [-20;30]	Sharma 2009 [108]
