# Supplementary material for: The protective effectiveness of control interventions for malaria prevention: a systematic review of the literature
Source: F1000Res. 2017 Nov 1;6:1932. [Version 1] doi: 10.12688/f1000research.12952.1 (PMC5721947; doi:10.12688/f1000research.12952.1)
Supplement: Supplementary file 6 [file f1000research-6-14045-s0005.tgz › 051bea8d-0615-46d6-bfd7-0f63dab71715.pdf]

**Supplementary File 5 – PE of IPTp.** PE of IPTp in decreasing order, by IPTp regimens compared, and by outcome measured. *: Indicates significant result. †: In multi-country studies the number between brackets indicates the number of countries. Std: Standard. CCS: case-control survey. CSS: cross-sectional survey.

| IPTp regimens compared | Outcome | Population | Study design | Country† | PE (%) [95%CI] | Reference |
|---|---|---|---|---|---|---|
| **Standard vs None** | Infection | Women | CSS | Burkina Faso | 94 [67;99]* | Gies 2009 [141] |
| **Standard vs None** | Infection | Women | Cohort | Uganda | 90 [82;94]* | De Beaudrap 2013 [71] |
| **Standard vs None** | Infection | Women | CSS | Burkina Faso | 70 [-50;90] | Cisse 2014 [41] |
| **Standard vs None** | Infection | Women | CSS | Uganda | 30 [10;60]* | Mbonye 2008 [143] |
| **Standard vs None** | Infection | Women | CSS | Uganda | 10 [-20;50] | Mbonye 2008 [143] |
| **Standard vs None** | Infection | Women | CSS | Gabon | 4 [-157;64] | Ramharter 2007 [145] |
| **Standard vs None** | Infection | Women | CSS | Cote d'Ivoire | -21 [-98;25] | Toure 2014 [146] |
| **Standard vs None** | Death | Infants | CSS | Multicountry (25) | 20 [10;30]* | Eisele 2012 [125] |
| **Standard vs None** | Obstetrical | Women | CSS | Cote d'Ivoire | 82 [68;90]* | Vanga-Bosson 2011 [130] |
| **Standard vs None** | Obstetrical | Women | CSS | Kenya | 50 [6;73]* | van Eijk 2004 [142] |
| **Standard vs None** | Obstetrical | Women | CSS | Malawi | 4 [-53;40] | Rogawski 2012 [144] |
| **Standard vs None** | Obstetrical | Women | CSS | Multicountry (25) | 25 [20;29]* | Eisele 2012 [125] |
| **Standard vs None** | Composite | Women | CSS | Cameroon | 0 [-100;58] | Tonga 2013 [137] |
| **Any vs None** | Infection | Women | CSS | Ghana | 82 [63;92]* | Wilson 2011 [147] |
| **Any vs None** | Infection | Women | CSS | Mozambique | 75 [75;76]* | Brentlinger 2007 [23] |
| **Any vs None** | Infection | Women | CSS | Burkina Faso | 51 [17;71]* | Sirima 2006 [151] |
| **Any vs None** | Infection | Women | CSS | Nigeria | 43 [9;65]* | Falade 2007 [153] |
| **Any vs None** | Infection | Women | CSS | Gabon | 30 [-110;80] | Bouyou-Akotet 2010 [52] |
| **Any vs None** | Infection | Women | CSS | Mali | -42 [-866;66] | Famanta 2011 [155] |
| **Any vs None** | Clinical | Women | CSS | Nigeria | 44 [35;51]* | Peter 2013 [152] |
| **Any vs None** | Clinical | Infants | Cohort | Tanzania | -131 [-388;-9]* | Harrington 2013 [156] |
| **Any vs None** | Obstetrical | Women | CSS | Tanzania | 70 [0;90]* | Mosha 2014 [148] |
| **Any vs None** | Obstetrical | Women | CSS | Nigeria | 68 [32;85]* | Bako 2009 [149] |
| **Any vs None** | Obstetrical | Women | CSS | Nigeria | 64 [34;80]* | Ezebialu 2012 [129] |
| **Any vs None** | Obstetrical | Women | CSS | Ghana | 62 [9;80]* | Hommerich 2007 [150] |
| **Any vs None** | Obstetrical | Women | CSS | Nigeria | 56 [-2;81] | Tongo 2011 [134] |
| **Any vs None** | Obstetrical | Women | CSS | Malawi | 50 [50;70]* | Gutman 2013 [131] |

| IPTp regimens compared | Outcome | Population | Study design | Country[†] | PE (%) [95%CI] | Reference |
|---|---|---|---|---|---|---|
| **Any vs None** | Obstetrical | Women | CSS | Tanzania | 30 [-41;66] | Nganda 2004 [132] |
| **Any vs None** | Obstetrical | Women | CSS | Tanzania | 7 [-72;49] | Harrington 2011 [154] |
| **Std vs Substandard** | Infection | Women | CSS | Cameroon | 82 [48;94]* | Anchang-Kimbi 2009 [157] |
| **Std vs Substandard** | Infection | Infants | Cohort | Cameroon | -96 [-257;-10]* | Apinjoh 2015 [162] |
| **Std vs Substandard** | Clinical | Women | Cohort | Burkina Faso | 50 [34;72]* | Valea 2012 [159] |
| **Std vs Substandard** | Obstetrical | Women | CSS | Tanzania | 69 [6;90]* | Mpogoro 2014 [135] |
| **Std vs Substandard** | Obstetrical | Women | CCS | Zambia | 67 [9;88]* | Mace 2015 [158] |
| **Std vs Substandard** | Obstetrical | Women | CSS | Burundi | 64 [59;69]* | Msyamboza 2007 [133] |
| **Std vs Substandard** | Obstetrical | Women | CSS | Uganda | 51 [-49;84] | Namusoke 2010 [136] |
| **Std vs Substandard** | Obstetrical | Women | CSS | DRC | 43 [15;62]* | Likwela 2012 [161] |
| **Std vs Substandard** | Obstetrical | Women | CSS | Gabon | 29 [-233;44] | Kurth 2010 [138] |
| **Std vs Substandard** | Composite | Women | CSS | Uganda | 48 [13;69]* | Arinaitwe 2013 [160] |
| **Std vs Substandard** | Composite | Women | CCS | Zambia | 24 [1;42]* | Mace 2015 [158] |
