# Supplementary material for: The protective effectiveness of control interventions for malaria prevention: a systematic review of the literature
Source: F1000Res. 2017 Nov 1;6:1932. [Version 1] doi: 10.12688/f1000research.12952.1 (PMC5721947; doi:10.12688/f1000research.12952.1)
Supplement: Supplementary file 7 [file f1000research-6-14045-s0006.tgz › fabdb07b-e543-419d-9564-86fd1a1213b5.pdf]

Supplementary File 6 – PE of domestic use of insecticides. PE of the domestic use of insecticides in decreasing order, by formulation, and by outcome. *: Indicates significant result. CCS: case-control survey. CSS: cross-sectional survey.

Formulation of insecticides	Outcome	Study design	Country	PE (%) [95%CI]	Reference
**Coils**	Clinical	CCS	Kenya	52 [-10;79]	Ernst 2009 [116]
**Coils**	Clinical	CCS	Kenya	40 [-10;70]	Siri 2010 [119]
**Coils**	Clinical	CCS	Indonesia	34 [-136;81]	Roosihermiatie 2000 [107]
**Coils**	Clinical	CCS	Malawi	-20 [-80;20]	Mathanga 2005 [97]
**Coils**	Clinical	CCS	Burkina Faso	-24 [-100;27]	Yamamoto 2009 [106]
**Coils**	Clinical	CCS	Indonesia	-24 [-521;75]	Roosihermiatie 2000 [107]
**Coils**	Clinical	CCS	Kenya	-85 [-359;25]	Ong'echa 2006 [111]
**Coils**	Clinical	CCS	Malawi	-120 [-250;-40]*	Mathanga 2005 [97]
**Coils**	Obstetrical	CSS	Nigeria	21 [-37;55]	Tongo 2011 [134]
**Sprays**	Infection	CSS	Cote d'Ivoire	17 [3;29]*	Houngbedji 2015 [78]
**Sprays**	Clinical	CCS	South Africa	47 [-2;73]	Coleman 2010 [117]
**Sprays**	Clinical	CCS	Yemen	38 [11;57]*	Al-Taiar 2009 [163]
**Sprays**	Clinical	CSS	Ethiopia	30 [10;50]*	Deressa 2007 [109]
**Sprays**	Clinical	CCS	Colombia	-102 [-249;-16]*	Alexander 2005 [96]
**Sprays**	Obstetrical	CSS	Nigeria	35 [-11;63]	Tongo 2011 [134]
**Repellents**	Infection	CSS	Tanzania	44 [-11;71]	Geissbühler 2009 [38]
**Repellents**	Clinical	CCS	India	-18 [-75;22]	Srinivas 2005 [164]
**Mixed**	Clinical	CCS	Kenya	43 [6;65]*	Snow 1998 [115]
**Mixed**	Clinical	CCS	Kenya	-16 [-57;15]	Snow 1998 [115]
**Mixed**	Clinical	CCS	Congo	-16 [-140;51]	Carme 1994 [120]
