# Supplementary material for: The protective effectiveness of control interventions for malaria prevention: a systematic review of the literature
Source: F1000Res. 2017 Nov 1;6:1932. [Version 1] doi: 10.12688/f1000research.12952.1 (PMC5721947; doi:10.12688/f1000research.12952.1)
Supplement: Peer review history

# Peer review: Referee response for version 1

**DOI:** 10.5256/f1000research.14045.r27512

**Published:** 2017-12-07

**Authors:** Christian Lengeler

**Affiliations:** 1 Swiss Tropical and Public Health Institute, Basel, Switzerland

The systematic review by Kesterman *et al.* is both timely and important. Measuring effectiveness of health interventions is important and increasingly done. Malaria control is fortunate to have many such studies done, especially with the strong development of secondary data analysis since the year 2000. But as shown very well by this review, the studies differ widely in their study designs, settings, age groups, and a myriad of other parameters. When reading publications on such studies it is often difficult to see the significance or generalizability of an individual study. With the present review, Kesterman *et al.* contribute greatly to show the range of studies and their results, and put some order in that vast body of information. By and large this is an excellent paper, well designed, researched and written up. The figures in particular are excellent.

 I have only one major issue, which is that their literature search stopped in 2015. For a paper submitted in November 2017, this is not really acceptable. Given that the analysis of the data is limited, an additional search and addition of the newest studies to the paper should not represent an enormous investment in time, and I would strongly suggest that this be done.

 Further, I have a number of small points, relating largely to the interpretation of the data and they are listed below:

 1. One more general reference on the difference between efficacy and effectiveness would be useful in the Introduction, at the end of para 2.This would help to clarify the difference for readers less familiar with these concepts. I have listed a few possible references below.

*JP Habicht et al. 1999. Evaluation designs for adequacy, plausibility and probability of public health programme performance and impact. International Journal of epidemiology 28; 10-18.*

*CG Victora et al. 2004. Evidence-based public health:  moving beyond randomized trials. American Journal of Public Health 94 (3), 400-405.*

*Tanner M, Lengeler C, Lorenz N.* *Case studies from the biomedical and health systems research activities of the Swiss Tropical Institute in Africa. Trans R Soc Trop Med Hyg. 1993 Sep-Oct;87(5):518-23.*

 2. Discussion para 1: The number of studies on effectiveness is stable since 2010, but at a high level. Malaria control interventions are really very well documented in comparison to many other health interventions. So the rather negative formulation that this could “hinder progress” is not really justified.

 3. Discussion para 3: a little more discussion on the risk of bias, which is substantial in the reviewed studies, would be useful.Certainly, the fact that some studies show a significantly increased risk (for which it is hard to find plausibility) is a good indication of how strong the bias can be.

 4. Discussion para 3: I agree in principle that these studies should guide local control activities. But given the risk of bias mentioned above, this should be really careful and guided by circumstances.

 5. Finally, in the discussion it would be good to re-visit briefly the point made in the introduction (3 ^rd^ para) that surveillance and ecological studies also provide data on the effect of malaria control. While it is to some extent correct that these tools look at the overall effect of different interventions and attribution is difficult, in practice in many settings only one preventive tool is applied with a high coverage - usually LLINs or IRS.  IPTp, case management and other interventions contribute probably little to impacting transmission, so surveillance can actually be a useful way to triangulate the results from specific studies.

I have read this submission. I believe that I have an appropriate level of expertise to confirm that it is of an acceptable scientific standard, however I have significant reservations, as outlined above.

# Peer review: Referee response for version 1

**DOI:** 10.5256/f1000research.14045.r27509

**Published:** 2017-11-24

**Authors:** Michel Cot

**Affiliations:** 1 Institut de Recherche pour le Développement (IRD), Mère et Enfant Face aux Infections Tropicales, 75006 Paris, France

This review reports on the effectiveness of malaria prevention interventions, worldwide. Whereas the subject of the efficacy of malaria control measures has been extensively assessed, the originality of this paper is to deliberately exclude efficacy studies (eg, randomized trials) and to focus on the protective effectiveness (PE) provided by control interventions under field conditions, such as case-control, cross-sectional surveys or cohort studies that are usually left aside. The context and the objectives are clearly laid out, and the methods are described in detail. Also, this considerable work addresses two important difficulties which have to be overcome, the high frequency of biases in non controlled designs, and the representativeness of such highly heterogeneous studies.  Indeed, they relate to very different interventions or populations (especially age groups which are adequately adjusted for, although they should not be the only ones), rendering the overall effect on PE difficult to interpret, so the cautious discussion on the final results is particularly precious.

 My major concern is that the search does not include references published after June 2015. In particular, the statement (Results, 3rd paragraph) that the number of publications related to the PE of interventions decreased since 2010, considering that some major studies were published in the last two years, is not true when performing a quick search in the databases. Some important results have been published in the period 2015 - 2017, such as a study on malaria prevention measures  on six Sub-Saharan African countries by Drakeley et al. ^1^; a complete issue of the American Journal of Tropical Medicine and Hygiene (2017, 97, issue 3 Suppl) with reports from several East African countries; a study on IRS in Northern Uganda ^2^. All these studies should be included in the review, as they may change the results (or at least, significantly contribute). Besides, the final paragraph of the discussion claims that the PE of SMC (seasonal malaria control) implemented in six African countries has not been evaluated. It was probably true  in 2015, but the evaluation has been done since, by Diawara et al. ^3^ in Mali, Cissé et al. ^4^ in Senegal, Tagbor et al. ^5^ in Ghana. These important results must definitely be included in the review, which would be incomplete otherwise.

 On the nature of the interventions that have been included, I agree that IPTp is a major intervention in the prevention of malaria and that IPTp studies in pregnant women should not be excluded from the review. However, the main outcome is generally different from parasitological or clinical indicators found in other studies, as it mainly consists in the measurement of the baby's birthweight (and the proportions of LBW babies). This particular outcome appears in the supplementary file 5, it should be more clearly specified in the text (results, and particularly in the methods where only two outcomes are mentioned).

I have read this submission. I believe that I have an appropriate level of expertise to confirm that it is of an acceptable scientific standard, however I have significant reservations, as outlined above.

## References

[1] DrakeleyCAbdullaSAgnandjiSTFernandesJFKremsnerPLellBMewonoLBacheBEMihayoMGJumaOTannerMTahitaMCTintoHDialloSLompoPD'AlessandroUOgutuBOtienoLOtienoSOtienoWOyiekoJAsanteKPDeryDBAdjeiGAdenijiEAtibillaDOwusu-AgyeiSGreenwoodBGesaseSLusinguJMahendeCMongiRSegejaMAdjeiSAgbenyegaTAgyekumAAnsongDBawaJTBoatengHODandaloLEscamillaVHoffmanIMaenjePMartinsonFCarterTLeboulleuxDKaslowDCUsufEPirçonJYBahmanyarER: Longitudinal estimation of Plasmodium falciparum prevalence in relation to malaria prevention measures in six sub-Saharan African countries. *Malar J*.2017;16(1) : 10.1186/s12936-017-2078-3 433 10.1186/s12936-017-2078-3 29078773PMC5658967

[2] OkulloAEMatovuJKBArioAROpigoJWanziraHOguttuDWKalyangoJN: Malaria incidence among children less than 5 years during and after cessation of indoor residual spraying in Northern Uganda. *Malar J*.2017;16(1) : 10.1186/s12936-017-1966-x 319 10.1186/s12936-017-1966-x 28784119PMC5547524

[3] DiawaraFSteinhardtLCMahamarATraoreTKoneDTDiawaraHKamateBKoneDDialloMSadouAMihigoJSagaraIDjimdeAAEckertEDickoA: Measuring the impact of seasonal malaria chemoprevention as part of routine malaria control in Kita, Mali. *Malar J*.2017;16(1) : 10.1186/s12936-017-1974-x 325 10.1186/s12936-017-1974-x 28797263PMC5553795

[4] CisséBBaEHSokhnaCNDiayeJLGomisJFDialYPittCNDiayeMCairnsMFayeENDiayeMLoATineRFayeSFayeBSyOKonateLKouevijdinEFlachCFayeOTrapeJFSutherlandCFallFBThiorPMFayeOKGreenwoodBGayeOMilliganP: Effectiveness of Seasonal Malaria Chemoprevention in Children under Ten Years of Age in Senegal: A Stepped-Wedge Cluster-Randomised Trial. *PLoS Med*.2016;13(11) : 10.1371/journal.pmed.1002175 e1002175 10.1371/journal.pmed.1002175 27875528PMC5119693

[5] TagborHAntwiGDAcheampongPRBart PlangeCChandramohanDCairnsM: Seasonal malaria chemoprevention in an area of extended seasonal transmission in Ashanti, Ghana: an individually randomised clinical trial. *Trop Med Int Health*.2016;21(2) : 10.1111/tmi.12642 224-35 10.1111/tmi.12642 26578353PMC4982104

# Peer review: Referee response for version 1

**DOI:** 10.5256/f1000research.14045.r27508

**Published:** 2017-11-13

**Authors:** Immo Kleinschmidt

**Affiliations:** 1 Department of Infectious Disease Epidemiology, London School of Hygiene & Tropical Medicine, London, UK

This is an important piece of work that examines the effectiveness of malaria control interventions in programmatic, non-research settings. Whilst such studies do not deliver the same quality of evidence as randomised trials, they provide important insights, and their sheer volume provides an understanding that we do not get from efficacy trials. This is particularly important for interventions like IRS, which have a wealth of programmatic evidence of effectiveness, but a dearth of randomised trials demonstrating impact. To see this body of work brought together and systematically synthesised in this paper is a great achievement indeed.

 Studies such as the ones that are included in this review are by their very nature somewhat messy and subject to bias and confounding. They therefore need to be interpreted with caution, and in relation to the available trial evidence, where this exists. This has largely been done in this review.

 Perhaps a bit more could have been said about adjustment for confounding other than by age, and the discussion could have mentioned the strong possibility of publication bias against non-significant findings.

 One group of studies that have not been included here are studies that compare post intervention outcomes with pre-intervention outcome in the same population. These studies are of course also methodologically flawed, but given the nature of this review I would have thought that some should have been eligible. This is particularly true for IRS studies where it is difficult to obtain measures of effect from cross-sectional surveys because the intervention acts at a community level, and because non-randomised contemporaneous control communities are as problematic as the before versus after design. Some studies have recorded such large changes in outcomes after the introduction of IRS, that it is unlikely that this is not associated with the intervention. Studies which represent an interrupted time series (for example Sharp *et al*, 2007) are particularly convincing in this regard. To me this review seems somewhat incomplete without their consideration. However, I fully accept that the authors may have had good reason for the exclusion of such studies from this review.

 A point to add to the discussion is the possibility of the impact of insecticide resistance on effectiveness, particularly in the case of IRS. A minor point is to use the term ‘susceptibility’ (of vectors to insecticide), rather than ‘sensitivity’ (presumably a translation issue).

I have read this submission. I believe that I have an appropriate level of expertise to confirm that it is of an acceptable scientific standard.
